# Phenolic Acid Composition and Anti-Parasitic Effects of Four* Peucedanum *Species on *Entamoeba histolytica* Trophozoites

**Published:** 2015

**Authors:** Serpil DEGERLI, Bektas TEPE

**Affiliations:** 1 *Department of Parasitology, School of Medicine, Cumhuriyet University, Sivas, Turkey*; 2 *Department of Molecular Biology and Genetics, Faculty of Science and Literature, Kilis 7 Aralik University, Kilis, Turkey *

**Keywords:** *Peucedanum caucasicum*, *Peucedanum palimbioides*, *Peucedanum longibracteolatum*, *Peucedanum chryseum*, *Entamoeba histolytica*, Phenolic acid

## Abstract

***Background:*** The aim of this study was to determine phenolic acid composition and anti-parasitic effects of *Peucedanum caucasicum, P. palimbioides, P. longibracteolatum *and* P. chryseum* on *Entamoeba histolytica*.

***Methods:*** Methanol extracts of the plant species were prepared by soxhlet extraction. Phenolic acid compositions were determined by HPLC. Anti-proliferative effect of extracts on trophozoites was determined by using trypan blue dye exclusion test. For counting the cells, approximately a hundred of *E. histolytica* trophozoites were examined in each time. The data were presented as mean values with standard deviations and analyzed by repeated measures of ANOVA followed by Tukey test for post-hoc pairwise comparisons. The *P*-value was set at 0.05 for significance level.

***Results:*** All of the extracts showed a time and dose dependent amoebicidal action on trophozoites. Among the extracts tested, *P. longibracteolatum* showed the strongest amoebicidal effect on the trophozoites. As expected, this plant species also exhibited time and dose dependent activity on the trophozoites. At 4.0 mg/ml extract concentration, all of the trophozoites were killed by the extract in 72^nd^ hour. Gallic (11.144 mg/g), *P*-hydroxybenzoic (17.646 mg/g), and *o*-coumaric acids (14.442 mg/g) were determined as the major phenolic acids of *P. longibracteolatum*. Gallic and *P*-hydroxybenzoic acids found in *P. longibracteolatum* could not be determined in other extracts. Therefore, high activity potential of this plant could probably be attributed to the presence of these phytochemicals.

***Conclusion:*** *P. longibracteolatum* can be further evaluated as potential therapeutic drugs for the treatment of *Entamoeba* infections.

## Introduction

Death from protozoan parasite, *Entamoeba histolytica*, is still one of the important health problems in developing countries that also known as third world countries. Among these deaths, amoebiasis takes the second place (1). It affects more than 10% of the world’s population. Untreated infections may cause to severe complications such as hepatic amoebiasis and intestinal tissue destruction (2). Amoebiasis is responsible for approximately 110,000 deaths worldwide annually. The main reason for about 50 million clinical cases each year is estimated as amoebiasis (3, 4).

*E. histolytica/dispar* is responsible for an estimated 35 to 50 million cases of disease and approximately 100 000 deaths annually (5). In general, most of morbidity and mortality are seen in Asia, Central and South America and Africa. 

 The most prevalence of amoebiasis was seen in 10-35 yr age groups globally. Children suffer from malnourishment and stunting as the result of infections occurs repeatedly (6). Infection resistance is directly related with the production of interferon-g and mucosal IgA produced by peripheral blood mononuclear cells. These agents are directed to the parasite surface lectin (7). Studies are underway to develop an effective vaccine. For treatment of invasive amoebiasis, limited numbers of agents mainly nitroimidazoles such as metronidazole are available today. However, toxic side effects can occur because of the treatment of infection with these drugs. Additionally, there is a need for additional drugs in 40–60% of patients to cure infection (8).

The prevalence of infection is known as due to the location and climate of the region where people live. However, it is closely related to sanitation and socioeconomic status of people rather than these factors. Poor life-style, environmental conditions of the tropics, and the non-availability of guaranteed conventional medical cure are among the complex etiological factors (9).

The genus *Peucedanum* L. (Apiaceae) is mainly distributed in Europe and Asia. It comprises about 100-120 species (10). *Peucedanum *species in Turkish flora are represented by 18 taxa (with 19 species, 2 of which are suspected). Among these species, seven are endemic to Turkey with an endemism rate of 28.5% (11-16). *Peucedanum* species are known as “kral out” in Turkey and they are under investigation due to their substantial amounts of chemically active phytochemicals, such as coumarins and their glycoside derivatives (10, 17, 18). Some members of this genus (*P. dissolutum* and *P. praeruptorum*) are used in traditional Chinese medicine (Qianhu) to cure ailments such as coughs, phlegm, and other respiratory illnesses (18, 19). Additionally, essential oil compositions, antimicrobial and antiproliferative activities of the some members of this genus have been investigated (18-20). 

Plant extracts can be thought as an alternative was to the classical chemical and/or synthetic methods for the treatment of parasitic infections. In this context, amoebicidal activities of some plant species have previously been reported by our research team (21-31). Drugs of natural origin have already been used to treat other parasitic diseases (32-35). 

The aim of this study was to determine in vitro susceptibility of *P. caucasicum, P. palimbioides, P. longibracteolatum *and* P. chryseum* for the treatment of gastrointestinal disorders. Additionally, determination of the phenolic acid composition of the samples was also aimed to have an idea about the relative contribution of these phytochemicals on anti-parasitic action. According to our literature survey, anti-parasitic activities of these plants have not previously been reported. Therefore, this study could be assumed as the first report on this topic.

## Materials and Methods

***Preparation of the methanol extracts***

The air-dried and ﬁnely ground samples were extracted by using a method described elsewhere (36). Brieﬂy, the sample, weighing about 100 g, was extracted in a Soxhlet apparatus with methanol (MeOH) at 60 ºC for 6 h. Due to the aqueous characteristics of the experimental media; mainly the water-soluble parts of the extracts could be used only. For obtaining the aqueous sub-fraction of the extracts, it was further fractionated with chloroform and distilled water. Finally, the extracts were then lyophilized and kept in the dark at +4 ºC until tested. Extract yields of the polar sub-fractions of the rhizome and aerial part samples were determined as 2.24%, 3.12%, 3.56%, and 3.40% w/w, respectively. 

***HPLC analysis***

The analysis of phenolic acids was employed according to the method described by Ozturk et al. (37) with a slight modification using an Agilent HPLC series 1200 (Agilent, Waldbronn, Germany). The separation of gallic (GA), protocathechuic (protoCA), *P*-hydroxy benzoic (*P*-hydBA), vanillic (VA), caffeic (CA), chlorogenic (ChA), syringic (SA), *P*-coumaric (*P*-COU), ferulic (FA), *o*-coumaric (*o*-COU), rosmarinic (RMA) and *trans*-cinnamic (*tr*-CIN) acids was performed on an Agilent Zorbax Eclipse XDB-C18 column (150 mm, 4.6 mm i.d., 5 µm particle size). The chromatographic conditions were: flow rate 1 mL / min, sample injection volume of 5 µL, operation temperature of 23 **°**C, UV detection at 280 nm and mobile phase A (methanol:water:formic acid (10:88:2, v/v)) and B (methanol: water:formic acid (90:8:2, (v/v)). A gradient program was used as follows: 100% A; 0 - 20 min, changed to 80% A; 25 - 50 min, to 50% A; 50 - 54 min, followed by isocratic elations of 50% A; 54 - 64 min, 0% A; and 64 - 70 min, 100% A. The results were evaluated with regard to the areas of the peaks and their retention times. Quantitation was based on calibration curves built for each of the compounds identified in the samples.

***Trophozoites***

*E. histolytica* strain was kindly provided by Dr. Graham Clark who is working in London School of Hygiene & Tropical Medicine and he is a specialist on amoebiasis. It was a pathogenic strain isolated from a Bangladeshi child with acute amoebic dysentery. Trophozoite forms of parasite were cultured monoxenically in the Robinson medium at 35.5 C° (37). Agar slants were prepared in screw-cap glass bijou bottles, 1.5 ml of phthalate, 1.5 ml of BRS medium, 50 μl of erythromycin and 100 μl Bacto peptone were mixed in the bottle and sterilized rice starch and *Escherichia coli* R medium are added to the medium before inoculation. Each time 0.5 ml of culture is used for inoculation and culture was renewed for a further 96 h to maintain the amoeba strain. The trophozoites in the log phase of growth were harvested by centrifugation at 500g at room temperature for 5 min. The pelleted cells were resuspended in Phosphate Buffer Saline (PBS), the final concentration was adjusted to 10x10^4 ^trophozoites/ml and the trophozoites were used in the assay immediately. The number of viable trophozoites was determined by trypan blue exclusion and direct trophozoite counts on a hemocytometer prior to experiment. 

***Determination the amoebicidal activity of plant extracts***

Eppendorf tubes were used in the present study. The plant extracts were dissolved and diluted in sterile distilled water to serial concentrations (2, 4, 8, 16, 32 and 64 mg/ml). Totally, 250 µl of the calibrated trophozoite suspension and the same volume of test solution were mixed thoroughly via pipetting up and down. Then the tubes were kept at 35.5 °C in standard bacteriological incubator (Electro-Mag) for 1, 2, 3, 6, 8, 24, 48 and 72 hours. The same procedure was applied to controls containing only trophozoite suspension and sterile distilled water. Cell growth was periodically monitored by light microscopy (Nikon, Eclipse E 200). 

Trypan Blue Dye Exclusion Test was used to determine the effect of extracts on the proliferation of parasite. Following the incubation periods at 35.5 °C, 25 μl parasite suspensions was simply mixed with the same volume of trypan blue (0.5 mg/ml) in counting chamber. The mixture was allowed to incubate 2-3 min at room temperature and then the unstained (viable) and stained (nonviable) cells were counted separately. Approximately a hundred of *E. histolytica* trophozoites were examined in each time, and all the tests were repeated three times. 

***Statistical analysis***

The data were presented as mean values with standard deviations and analyzed by repeated measures of ANOVA followed by Tukey test for post-hoc pairwise comparisons. The *P*-value was set at 0.05 for significance level.

## Results

In vitro amoebicidal activity of the polar extract of *P. caucasicum* extract on *E. histolytica* trophozoites is presented in Table 1. *O. syriacum* extract showed the weakest trophozoidal activity in this study. At 32.0 mg/ml concentration, within 72^nd^ hour, approximately, 60% of the trophozoites were killed by the extract. 

Susceptibility of *E. histolytica* trophozoites to the polar extract of *P. palimbioides* is also presented in Table 2. *P. palimbioides* extract showed remarkable trophozoidal activity. At 16.0 mg/ml concentration, the extract killed 50% of the trophozoites in 8^th^ hour. At this concentration, within the 72^nd^ hour, approximately 80% of the trophozoites were killed. At 32.0 mg/ml concentration, number of the viable trophozoites was 10% within 72^nd^ hour. 

**Fig. 1:**
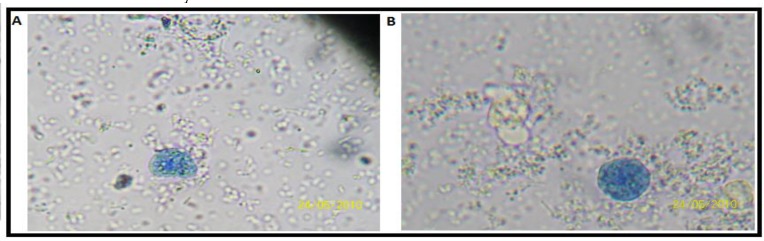
^A^ *Entamoeba histolytica* trophozoites treated with 4.0 mg/ml *P. longibracteolatum* extract in 72^nd^ hour  ^B^ *Entamoeba histolytica* trophozoites treated with 4.0 mg/ml *P. longibracteolatum* extract in 48^th^ hour

One another plant species of which amoebicidal activity investigated was *P. longibracteolatum *(Fig. 1). Polar methanolic extract obtained from this plant species was applied to *E. histolytica* trophozoites (Table 3). This extract showed the strongest activity potential on the trophozoites. As expected, this plant species also exhibited time and dose dependent activity on the trophozoites. At 4.0 mg/ml extract concentration, all of the trophozoites were killed by the extract in 72^nd^ hour. The extract at 8.0 mg/ml concentration was also killed all of the trophozoites in 48^th^ hour. In the case of 16.0 and 32.0 concentrations, after 8^th^ hour, no viable trophozoites were observed in the media.

Finally, in vitro amoebicidal activity of the polar methanol extract of *P. chryseum* was investigated on *E. histolytica* trophozoites (Table 4). The extract at 32.0 mg/ml, exhibited great activity potential. At 32.0 mg/ml concentration, more than 50% of the trophozoites were killed effectively in 24^th^ hour. At the end of the experimental process, the number of the viable trophozoites was determined as 15.3% at the same concentration level.

(Optical microscope magnifications 400X)

**Table 1:** Effect of *P. caucasicum* on the proliferation of *E. histolytica* trophozoites a

**Dose (mg/ml)**	**Experimental Period** **(Number of viable trophozoites, %)**						
	**1. hour**	**3. hour**	**6. hour**	**8. hour**	**24. hour**	**48. hour**	**72. hour**
32.0	77.3 ± 1.5 a	70.7 ± 2.9^ b^	64.7 ± 2.1 ^c^	62.3 ± 2.1 ^c^	54.3 ± 1.2 ^d^	48.3 ± 2.9 ^e^	38.0 ± 2.0 ^f^
16.0	87.3 ± 1.2 a	80.7 ± 1.5 ^b^	76.0 ± 2.0 ^c^	68.7 ± 0.6 ^d^	62.7 ± 1.2 ^e^	54.7 ± 1.2 ^f^	47.0 ± 1.7 ^g^
8.0	89.3 ± 0.6 a	86.3 ± 1.5 ^b^	85.7 ± 2.9 ^b^	83.3 ± 2.5 ^b^	77.3 ± 2.9 ^c^	68.7 ± 0.6 ^d^	57.7 ± 1.5 ^e^
4.0	90.0 ± 1.0 a	88.7 ± 1.5 a	87.3 ± 1.2 a	85.3 ± 1.2 ^b^	78.0 ± 1.7 ^c^	73.7 ± 2.5 ^d^	64.7 ± 1.2 ^e^
2.0	92.3 ± 1.5 a	90.7 ± 1.5 a	89.0 ± 1.0 ^b^	87.0 ± 1.0 ^b^	82.0 ± 1.7 ^c^	76.7 ± 0.6 ^d^	73.7 ± 0.6 ^e^
1.0	92.7 ± 1.2 a	91.3 ± 2.1 a	90.0 ± 1.0 a	88.3 ± 0.6 ^b^	83.3 ± 0.6 ^c^	78.7 ± 1.2 ^d^	75.0 ± 1.7 ^d^
Control	93.7 ± 1.2 a	92.3 ± 2.1 ^b^	91.7 ± 1.5 ^b^	91.0 ± 1.0 ^b^	89.7 ± 1.5 ^c^	87.3 ± 0.6 ^d^	85.0 ± 2.0 ^d^

Each row was evaluated individually from the statistical point of view.

a Date were expressed as mean ± SD.

**Table 2:** Effect of *P. palimbioides* on the proliferation of *E. histolytica* trophozoites a

**Dose (mg/ml)**	**Experimental Period** **(Number of viable trophozoites, %)**						
	**1. hour**	**3. hour**	**6. hour**	**8. hour**	**24. hour**	**48. hour**	**72. hour**
32.0	80.7 ± 1.2 a	73.7 ± 1.5 ^b^	57.3 ± 2.5 ^c^	47.7 ± 2.5 ^d^	38.3 ± 3.2 ^e^	17.7 ± 2.5 ^f^	10.0 ± 1.7 ^g^
16.0	85.0 ± 1.7 a	76.3 ± 1.5 ^b^	65.3 ± 1.2 ^c^	48.7 ± 2.3 ^d^	44.7 ± 3.5 ^e^	24.3 ± 1.5 ^f^	20.7 ± 1.2 ^g^
8.0	87.3 ± 2.1 a	78.3 ± 2.9 ^b^	67.7 ± 2.5 ^c^	55.7 ± 3.1 ^d^	50.0 ± 3.6 ^e^	33.3 ± 1.5 ^f^	28.3 ± 1.5 ^g^
4.0	88.0 ± 2.0 a	80.7 ± 1.2 ^b^	72.7 ± 1.2 ^c^	58.0 ± 2.6 ^d^	51.3 ± 1.5 ^e^	41.3 ± 1.2 ^f^	36.0 ± 1.7 ^g^
2.0	90.0 ± 0 a	82.3 ± 2.5 ^b^	75.0 ± 2.0 ^c^	67.3 ± 3.1 ^d^	64.7 ± 1.5 ^d^	60.7 ± 1.2 ^e^	54.3 ± 2.1 ^f^
1.0	92.3 ± 0.6 a	87.7 ± 2.1 ^b^	81.7 ± 1.5 ^c^	75.3 ± 0.6 ^d^	73.3 ± 2.1 ^d^	69.0 ± 2.6 ^e^	63.3 ± 1.5 ^f^
Control	96.0 ± 1.7 a	95.7 ± 2.1 a	93.3 ± 2.1 a	92.3 ± 0.6 ^b^	91.7 ± 1.5 ^b^	89.7 ± 0.6 ^c^	89.0 ± 1.7 ^c^

Each row was evaluated individually from the statistical point of view

a Date were expressed as mean ± SD.

**Table 3:** Effect of *P. longibracteolatum* on the proliferation of *E. histolytica* trophozoites a

**Dose (mg/ml)**	**Experimental Period** **(Number of viable trophozoites, %)**						
	**1. hour**	**3. hour**	**6. hour**	**8. hour**	**24. hour**	**48. hour**	**72. hour**
32.0	73.0 ± 2.6 a	55.7 ± 3.2 ^b^	26.3 ± 1.2 ^c^	13.3 ± 2.5 ^d^	0 ^e^	0 ^e^	0 ^e^
16.0	74.3 ± 2.1 a	64.7 ± 1.5 ^b^	50.7 ± 1.2 ^c^	27.7 ± 2.3 ^d^	0 ^e^	0 ^e^	0 ^e^
8.0	79.0 ± 1.0 a	72.3 ± 3.2 ^b^	60.3 ± 0.6 ^c^	48.3 ± 1.5 ^d^	24.7 ± 1.5 ^e^	0 ^f^	0 ^f^
4.0	87.0 ± 1.7 a	77.3 ± 2.5 ^b^	71.3 ± 1.5 ^c^	61.0 ± 1.7 ^d^	44.3 ± 1.2 ^e^	28.0 ± 2.0 ^f^	0 ^g^
2.0	89.7 ± 1.5 a	84.7 ± 0.6 ^b^	77.3 ± 0.6 ^c^	73.3 ± 1.2 ^d^	71.3 ± 1.5 ^d^	63.0 ± 2.6 ^e^	51.3 ± 2.5 ^f^
1.0	91.0 ± 1.7 a	88.7 ± 2.3 ^b^	87.3 ± 2.3 ^b^	83.3 ± 1.5 ^c^	79.0 ± 2.6 ^d^	70.0 ± 2.0 ^e^	65.7 ± 1.2 ^f^
Control	92.7 ± 1.2 a	92.3 ± 2.1 a	92.0 ± 2.0 a	90.3 ± 0.6 ^b^	89.7 ± 0.6 ^b^	89.3 ± 1.5 ^b^	88.7 ± 1.2 ^c^

Each row was evaluated individually from the statistical point of view

a Date were expressed as mean ± SD.

**Table 4:** Effect of *P. chryseum* on the proliferation of *E. histolytica* trophozoites a

**Dose (mg/ml)**	**Experimental Period** **(Number of viable trophozoites, %)**						
	**1. hour**	**3. hour**	**6. hour**	**8. hour**	**24. hour**	**48. hour**	**72. hour**
32.0	82.3 ± 0.6 a	76.3 ± 1.2 ^b^	61.7 ± 2.9 ^c^	53.0 ± 2.6 ^d^	42.7 ± 2.5 ^e^	23.0 ± 1.7 ^f^	15.3 ± 1.5 ^g^
16.0	85.3 ± 1.5 a	79.7 ± 1.5 ^b^	72.7 ± 1.5 ^c^	66.3 ± 2.1^ d^	54.7 ± 2.9 ^e^	45.3 ± 1.5 ^f^	31.3 ± 1.2 ^g^
8.0	91.3 ± 1.2 a	84.0 ± 2.6 ^b^	78.7 ± 2.3 ^c^	74.3 ± 2.5 ^d^	67.0 ± 2.0 ^e^	56.0 ± 2.6 ^f^	46.3 ± 1.2 ^g^
4.0	92.0 ± 2.0 a	90.7 ± 1.0 a	85.7 ± 2.5 ^b^	82.3 ± 1.5 ^b^	75.7 ± 1.2 ^c^	59.7 ± 2.5 ^d^	59.3 ± 2.5 ^d^
2.0	92.3 ± 2.5 a	91.0 ± 1.7 a	88.0 ± 2.0 ^b^	82.7 ± 2.1 ^c^	79.0 ± 1.0 ^d^	72.7 ± 2.5 ^e^	67.3 ± 1.5 ^f^
1.0	93.0 ± 1.0 a	92.3 ± 2.1 a	90.0 ± 2.6 a	86.3 ± 2.1 ^b^	83.0 ± 1.0 ^c^	80.0 ± 1.7 ^d^	77.7 ± 2.1 ^e^
Control	96.3 ± 1.2 a	92.7 ± 0.6 ^b^	92.3 ± 0.6 ^b^	91.7 ± 1.5 ^b^	90.7 ± 1.2 ^c^	90.0 ± 1.0 ^c^	89.0 ± 1.7 ^c^

Each row was evaluated individually from the statistical point of view

a Date were expressed as mean ± SD.

**Fig. 2:**
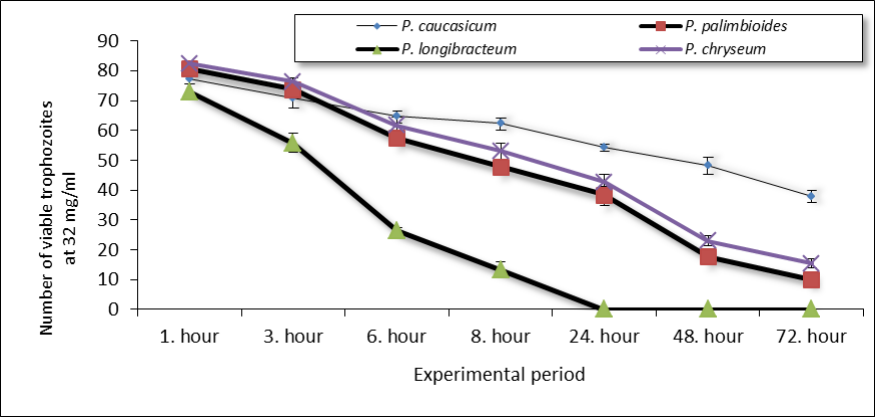
Effect of *Peucedanum *species on the proliferation of *E. histolytica* trophozoites at 32.0 mg/ml concentration (figure shows the number of viable trophozoites)

For making a better comparison between the results presented in Tables 1-4, data obtained from the 32.0 mg/ml concentration was also presented in Fig. 2.

By this study, we also reported the strongest amoebicidal effect of *P. longibracteolatum* on the trophozoites and cysts. From the twenty-fourth hour, no viable trophozoites or cysts were determined at 32 mg/ml of extract concentration. Similar results were also obtained with the extract at 16.0 mg/ml concentration. At this concentration, number of viable cysts was determined as 10.6 from the twenty-fourth hour of experimental process. At 8.0 mg/ml extract concentration, 51% of the cysts were killed by the extract in 72^nd^ hour.

**Fig. 3:**
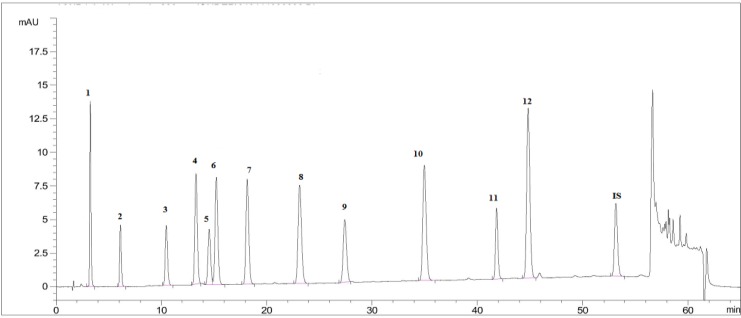
HPLC chromatogram of the standard phenolic acids used in the study (The name of peaks corresponding to the numbers; 1: gallic acid, 2: protocatechuic acid, 3: *P*-hydroxybenzoic acid, 4: vanillic acid, 5: caffeic acid, 6: chlorogenic acid, 7: syringic acid, 8: *P*-coumaric acid, 9: ferulic acid, 10: rosmarinic acid, 11: *o*-coumaric acid, 12: *trans*-cinnamic acid, IS: Internal standard)

**Fig. 4:**
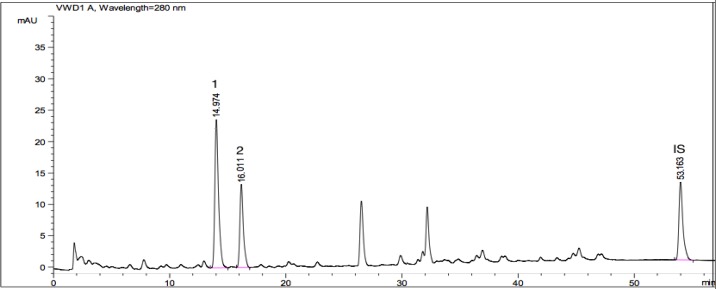
HPLC chromatogram showing phenolic acids available in *P. caucasicum* A (1: vanillic acid (7.167 mg/g), Rt (minute): 14.974, Source of confirmation: UV, commercial standard; 2: chlorogenic acid (4.885 mg/g), Rt (minute): 16.011, Source of confirmation: UV, commercial standard; IS: Internal Standard, Rt (minute): 53.163)

**Fig. 5:**
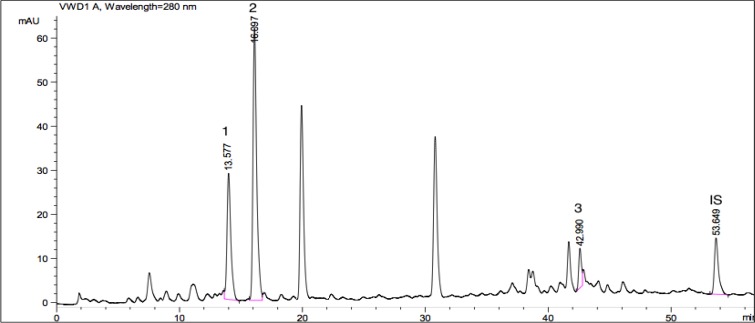
HPLC chromatogram showing phenolic acids available in *P. palimbioides* A (1: vanillic acid (14.640 mg/g), Rt (minute): 13.577, Source of confirmation: UV, commercial standard; 2: chlorogenic acid (29.215 mg/g), Rt (minute): 16.097, Source of confirmation: UV, commercial standard; 3: rosmarinic acid (6.577 mg/g), Rt (minute): 42.990, Source of confirmation: UV, commercial standard; IS: Internal Standard, Rt (minute): 53.649)

**Fig. 6:**
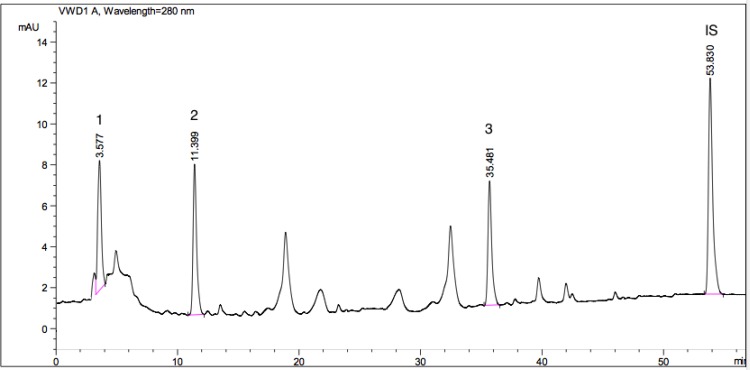
HPLC chromatogram showing phenolic acids available in *P. longibracteolatum* A (1: gallic acid (11.144 mg/g), Rt (minute): 3.577, Source of confirmation: UV, commercial standard; 2: *p*-hydroxybenzoic acid (17.646 mg/g), Rt (minute): 11.399, Source of confirmation: UV, commercial standard; 3: *o*-coumaric acid (14.442 mg/g), Rt (minute): 35.481, Source of confirmation: UV, commercial standard; IS: Internal Standard, Rt (minute): 53.830)

**Fig. 7:**
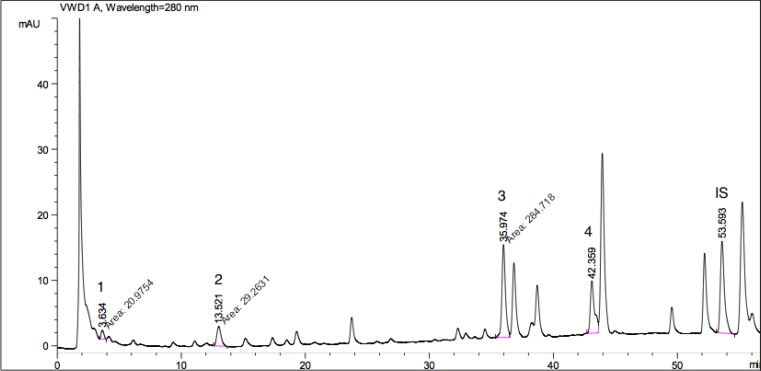
HPLC chromatogram showing phenolic acids available in *P. chryseum* A (1: gallic acid (1.347 mg/g), Rt (minute): 3.634, Source of confirmation: UV, commercial standard; 2: vanillic acid (3.014 mg/g), Rt (minute): 13.521, Source of confirmation: UV, commercial standard; 3: *o*-coumaric acid (8.420 mg/g), Rt (minute): 35.974, Source of confirmation: UV, commercial standard; 4: rosmarinic acid (4.642 mg/g), Rt (minute): 42.359, Source of confirmation: UV, commercial standard; IS: Internal Standard, Rt

Apart from the anti-parasitic activities, plant species evaluated here are also screened for their phenolic acid compositions. By this way, we tried to have an idea about the relative contribution of these phytochemicals in anti-parasitic potential of extracts. For this purpose, twelve different phenolic acids including; gallic acid, protocatechuic acid, *P*-hydroxybenzoic acid, vanillic acid, caffeic acid, chlorogenic acid, syringic acid, *P*-coumaric acid, ferulic acid, rosmarinic acid, *o*-coumaric acid, *trans*-cinnamic acid were screened within the extracts. Peaks of standard phenolic acids used are given as a chromatogram in Fig. 3. HPLC chromatograms of the plant species showing phenolic acid compositions are presented as Fig. 4-7.

Vanillic, chlorogenic, rosmarinic, gallic, *P*-hydroxybenzoic, and *o*-coumaric acids were determined within the extracts. The most common phenolic acid was determined as vanillic acid that found in *P. caucasicum*, *P. palimbioides*, and *P. chryseum*. As seen from Table 3, *P. longibracteolatum* showed the highest anti-parasitic activity against *E. histolytica* trophozoites. According to HPLC analysis of this sample, gallic (11.144 mg/g), *P*-hydroxybenzoic (17.646 mg/g), and *o*-coumaric acids (14.442 mg/g) were determined as the major phenolic acids (Fig. 6). Plant containing the maximum number of phenolic acids was *P. chryseum* of which main compounds were determined as gallic (1.347 mg/g), vanillic (3.014 mg/g), *o*-coumaric (8.420 mg/g), and rosmarinic acids (4.642 mg/g) (Fig. 7). As can be seen from Table 2, anti-parasitic activity of *P. longibracteolatum* was followed by *P. palimbioides*. According to chromatographic analyses, a similar phenolic acid profile was observed for this plant (Fig. 5). 

The main phenolic acids of this plant were determined as vanillic (14.640 mg/g), chlorogenic (29.215 mg/g), and rosmarinic acids (6.577 mg/g). Phenolic composition of *P. caucasicum* was also determined chromatographically (Fig. 4). Vanillic (7.167 mg/g) and chlorogenic acids (4.885 mg/g) were found as the major phenolic acids among the standards screened.

## Discussion

Enteric protozoan infections are among the important causes of morbidity and mortality. They are still known as the serious health problems in developing countries. Abdominal pain and tenderness, diarrhea, and bloody stools are among the most common symptoms frequently observed in patients having an *Entamoeba *infection. By the treatment amoebiasis, eliminating clinical symptoms and destroying amoeba in its cyst or trophozoite forms are aimed. Amoebicides are frequently administered to the patients orally, intramuscularly and intravenously. Currently, in the treatment of amoebic infections such as dysentery and liver abscesses, just a few amoebicides can be used due to their narrow therapeutic uses and undesirable side effects on patients. In dysentery, metronidazole is the main drug of choice. It is known as a highly effective agent against *Entamoeba*. However, this drug has mutagenic effects in bacteria and is carcinogenic in some animals. On the other hand, headache, dry mouth, a metallic taste, and neurotoxicity and gastrointestinal disturbances, especially nausea is among the most common side effects of metronidazole. In some patients, vomiting and diarrhoea or constipation may also occur (38, 39). *E. histolytica* can show multidrug resistance during the treatment (40). Metronidazole resistance was induced experimentally in the *E. histolytica* axenic strain HTH-56: MUTM (41).

Plant extracts possess a wide array of biological activities that can be easily used for the betterment of human beings. Many plants, used in traditional medicine, proved to be of immense importance and gave significant results in having antiamoebic activities. A detailed study of plants with antiamoebic properties should be done based on the ethnobotanical leads available. However, these findings can only be of practical use when they would undergo stringent clinical trials (42). 

Plant species presented here were also investigated on *Acanthamoeba castellanii* trophozoites and cysts by our research team (25). Our research group is currently focused on the anti-parasitic effects of plant species growing wild in Turkey. Within this project amoebicidal effect of several plant species were identified (22-31). 

Among the extracts tested, *P. longibracteolatum* showed the strongest amoebicidal effect on the trophozoites (Fig. 1).. As expected, this plant species also exhibited time and dose dependent activity on the trophozoites. At 4.0 mg/ml extract concentration, all of the trophozoites were killed by the extract in 72^nd^ hour. The extract at 8.0 mg/ml concentration was also killed all of the trophozoites in 48^th^ hour. 

Materials extracted with methanol were further fractionated with chloroform and water to obtain polar and non-polar phytochemicals separately. In experimental process presented here, it is necessary using the polar components to determine their amoebicidal activities due to the polarity of media that requires the using polar substances. Polar compounds can enter chemical reactions easier than those of non-polar ones within the cells can. By this way, they can show desired activity potential more effectively. 

In general, we could not establish a direct relationship between phenolic acid composition and anti-parasitic activity potential of plants. However, as can be seen from the chromatograms, gallic and *P*-hydroxybenzoic acids mainly found in *P. longibracteolatum* extract could not be determined in other extracts. Therefore, high activity potential of this plant could probably be attributed to the presence of these phytochemicals. However, in order to have a clearer judgment on this topic, anti-parasitic activities of individual phenolic acid components should also be evaluated.

## Conclusion

*P. longibracteolatum* showed strongest activity potential on the proliferation of *E. histolytica* trophozoites. This activity was followed by *P. palimbioides, P. chryseum, *and *P. caucasicum*, respectively. These results suggest that *P. longibracteolatum* can be further evaluated as potential therapeutic drugs for the treatment of *Entamoeba* infections. 
